# Aptamer-Assisted Proximity Ligation Assay for Sensitive Detection of Infectious Bronchitis Coronavirus

**DOI:** 10.1128/spectrum.02081-22

**Published:** 2023-01-18

**Authors:** Issam Hmila, Boutheina Marnissi, Masood Kamali-Moghaddam, Abdeljelil Ghram

**Affiliations:** a Laboratory of Epidemiology and Veterinary Microbiology, Institute Pasteur of Tunis, University of Tunis El Manar, Tunis, Tunisia; b Department of Immunology, Genetics and Pathology, Uppsala University, Uppsala, Sweden; c Science for Life Laboratory, Uppsala University, Uppsala, Sweden; University of Georgia

**Keywords:** aptamer, detection, infectious bronchitis coronavirus, proximity ligation assay, SELEX

## Abstract

Infectious bronchitis virus (IBV) is a coronavirus responsible for major health problems in the poultry industry. New virus strains continue to appear, causing large economic losses. To develop a rapid and accurate new quantitative assay for diagnosis of the virus without DNA extraction, we selected highly specific single-stranded DNA (ssDNA) aptamers with a high affinity to IBV, using the systematic evolution of ligands by exponential enrichment (SELEX) technology for aptamer screening, followed by high-throughput sequencing technology. Two of these aptamers, AptIBV5 and AptIBV2, were used to establish homogenous and solid-phase proximity ligation assays (PLAs). The developed assays were evaluated for their sensitivity and specificity using collected field samples and then compared to the newly developed sandwich enzyme-linked aptamer assay (ELAA) and reverse transcription-quantitative PCR (qRT-PCR), as the gold-standard method. The solid-phase PLA showed a lower limit of detection and a broader dynamic range than the two other assays. The developed technique may serve as an alternative assay for the diagnosis of IBV, with the potential to be extended to the detection of other important animal or human viruses.

**IMPORTANCE** Infectious bronchitis virus (IBV) causes high morbidity and mortality and large economic losses in the poultry industry. The virus has the ability to genetically mutate into new IBV strains, causing devastating disease and outbreaks. To better monitor the emergence of this virus, the development of a rapid and highly sensitive diagnostic method should be implemented. For this, we generated aptamers with high affinity and specificity to the IBV in an ssDNA library. Using two high-affinity aptamers, we developed a sandwich ELAA and a very sensitive aptamer-based proximity ligation assay (PLA). The new assay showed high sensitivity and specificity and was used to detect IBV in farm samples. The PLA was compared to the newly developed sandwich ELAA and qRT-PCR, as the gold-standard technique.

## INTRODUCTION

Infectious bronchitis (IB) disease continues to be a major problem for the poultry industry due to its high morbidity and mortality and the associated production losses. It is ubiquitous in most parts of the world where poultry is reared and can spread very rapidly in unprotected birds.

Infectious bronchitis virus (IBV) belongs to the order Nidovirales, family *Coronaviridae*, subfamily *Coronavirinae*, genus *Gammacoronavirus* (1). IBV has a nonsegmented, single-stranded, positive-sense RNA genome of >27 kb (2, 3). The genome encodes four major structural proteins. The nucleocapsid (N) protein (conserved across various IBV strains) is surrounded by an envelope in which the large spike (S) glycoprotein, as a dimer or a trimer, plays a determinant role in the interaction with the host cell (4, 5). The S protein is initially translated into a precursor glycoprotein that is cleaved posttranslationally to form two subunits, S1 (approximately 520 amino acids [aa]) and S2 (approximately 625 aa) (6). Each subunit has a different function. While the N-terminal S1 subunit is involved in attachment to the cellular receptors (7), the C-terminal S2 subunit anchors S1 to the viral envelope. According to Y. Yamada and D. X. Liu (8), S1/S2 cleavage could promote syncytium formation and infectivity of IBV in Vero cells (8). The S protein is involved in hemagglutination-inhibiting antibody production (7, 9) and is the main inducer of protective immunity (7, 10). This protein is also used to determine the virus serotype and is a vaccine component. The virus also contains a smaller integral membrane (M) protein and a few copies of a much smaller envelope (E) protein.

IBV primarily targets the ciliated epithelial cells of the respiratory tract (nose, trachea, lungs, and air sacs), causing a respiratory disease that predisposes the bird to secondary bacterial infections. Some IBV strains, known as nephron pathogenic, target the tubular cells of the kidneys. Infection with such strains can result in interstitial nephritis and significant mortality (11). Many strains also infect the female reproductive tract (oviduct), causing egg drops and “false layer” syndrome, attributed to a highly virulent strain (strain QX) that infects very young chickens. IBV may also affect the intestinal tract; in addition, Harderian gland cases of a proventricular form were also reported (12, 13).

The ability of IBV to genetically mutate and recombine results in antigenic shift and drift related to RNA recombination, mutations, insertions, and/or deletions, which has enabled the emergence of large numbers of IBV variants worldwide (14). Multiple distinct genetic groups of IBV have been reported, and many new variants continued to be isolated. There has been an increasing number of new serotype variants of IBV, and to date, more than 20 IBV serotypes have been identified. A change of only a small percentage of amino acids in the S1 protein may result in a serotype change (15). Outbreaks of IBV frequently occur following the persistence of different IBV serotypes among susceptible birds. Since the year 2000, our laboratory has isolated several novel IBV variants from the field which were cocirculating with Massachusetts H120, the only vaccine strain used in Tunisia before the introduction of closely related variant vaccine strains (16–18). These new strains with new genotypes have caused large economic losses to the poultry industry and thus should be controlled.

Current IBV diagnostic methods include virus isolation in chicken embryos, inoculation of cell and/or organ cultures, virus neutralization, reverse transcription-PCR (RT-PCR), an agar gel precipitation test, and an antigen-capture enzyme-linked immunosorbent assay (ELISA). However, these conventional methods are time-consuming, laborious, and less suitable for rapid and routine detection of IBV. There is a need for the development of a more rapid and convenient quantitative test for viral detection with minimal sample preparation. For this reason, we developed aptamer-based tools for rapid diagnosis of IBV, since aptamers have been investigated as alternative biorecognition ligands and have become one of the most promising nanomolecules in medicine. Their application has been widely extended, as therapeutic molecules, for the development of biosensors, or for the specific delivery of active molecules (19–21). In fact, they are functional short chains of nucleic acids of 20 to 90 bases. Their folding into a variety of secondary structures offers a large area for antigen recognition and allows them to be powerful agents for targeting and binding to any molecules. Their specific recognition of target molecules and high affinity to nanomolar or subnanomolar ranges (20) make them powerful tools for biorecognition. Furthermore, one of the major advantages of aptamers is the *in vitro* selection method, using selective ligands by exponential enrichment (SELEX), from a single-stranded DNA (ssDNA) library of up to 10^15^ randomized sequences. SELEX is a robust and straightforward *in vitro* method allowing the selection of highly specific aptamers with high affinity. Furthermore, the choice of aptamers as ligands allows signal amplification, yielding a highly sensitive detection test. The combination of aptamers as ligands with a proximity ligation assay (PLA) was investigated for more sensitive detection of IBV. PLA is a versatile and powerful technology for the detection, localization, and quantification of proteins, protein-protein interactions, and posttranslational modifications in liquid biopsy specimens, as well as *in situ* (22–25).

In the present work, we report the generation and characterization of ssDNA aptamers with high affinity and specificity to IBV. We then developed two rapid and efficient aptamer-based PLAs for IBV detection in farm samples. The efficiency of these aptamer-based PLA methods was compared to the newly developed sandwich enzyme-linked aptamer assay (ELAA) and to reverse transcription-quantitative PCR (qRT-PCR), as the gold-standard technique.

## RESULTS

### Generation of aptamers against IBV.

The ssDNA aptamers against IBV were generated using NaCl elution-based SELEX (26, 27), with three rounds of selection, and the ssDNA pool was sequenced using high-throughput sequencing. The DNA sequence pools were analyzed using FASTAptamer software. We chose the five first clusters as the most abundant sequences in the DNA output pool. The obtained sequences with their ranks and number of reads are summarized in Table 1.

**TABLE 1:** Sorted sequences based on FASTAptamer-Cluster and the binding affinities of the selected aptamers

Sequence (5′–3′)	Rank of sequence	No. of reads	RPM^*a*^	Cluster no.	*K_d_* (nM)^*b*^
CCCCAATCACAGTTAATCCTCGTTCCTATATCTCCCACAC	1	2,416	132.3	1	99.25
CACGTCTCTCTATTCGCTCCCTTCGCTAATTGTTCTCTCC	2	2,113	115.7	2	58.2
CAGACTTTGTTCAGCTCCTCGACTCTTCATTCCCTCCCTT	3	1,943	106.4	3	108.61
CCAGTATCATCCCTATCGTAGTCCTCAACAACCCCCTACA	4	1,898	103.93	4	99.94
GACTTCCCTCTTTGTGTGTCATTCGGTGTTCTCGCTTTTG	5	1,877	102.78	5	59.42

a RPM, number of reads per million.

b *K_d_*, dissociation constant; nM, dissociation constant in nanomolar.

### Binding affinity of aptamers.

**FIG 1:**
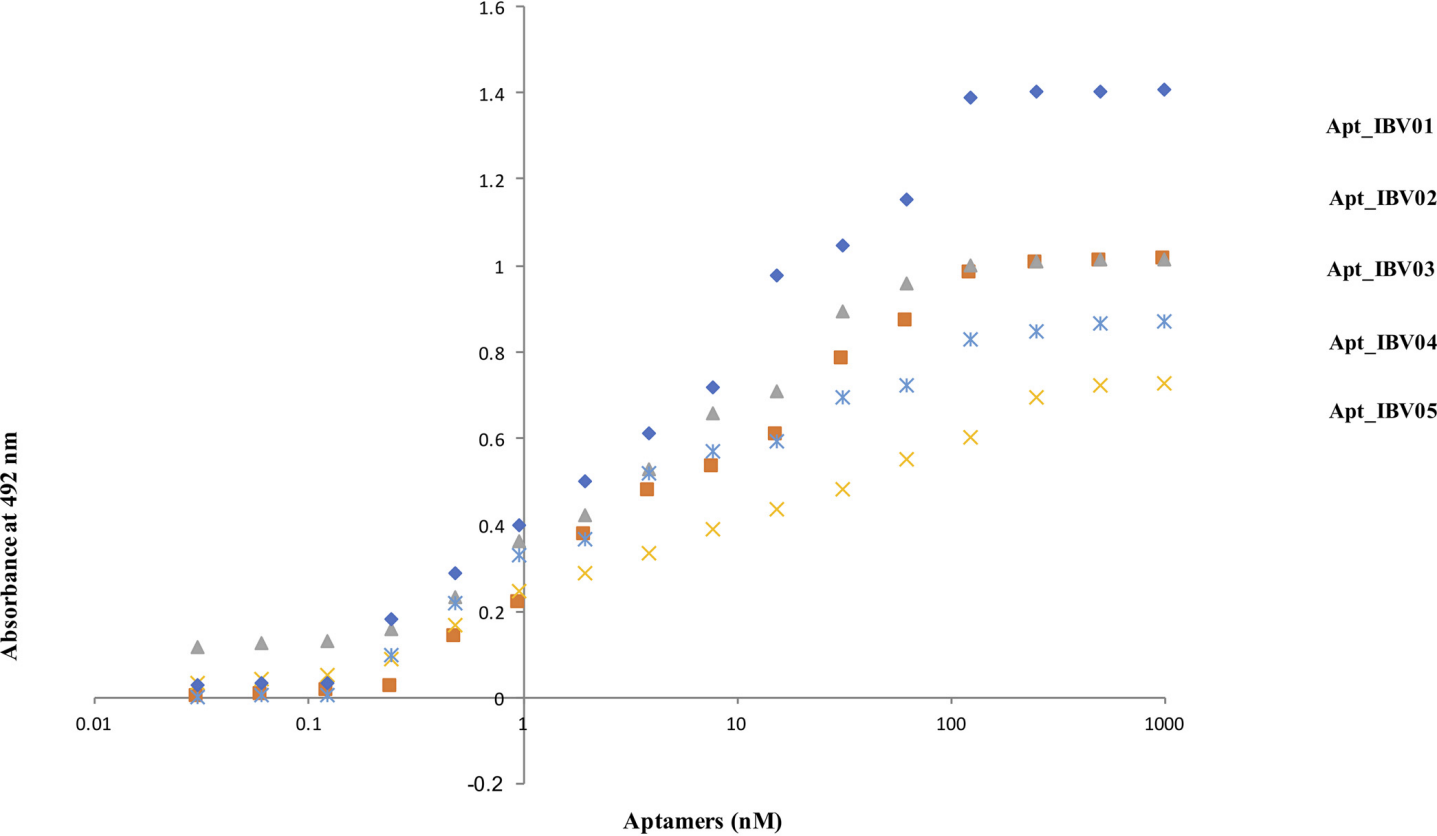
Dissociation constants (*K_d_*) at the lowest nanomolar range for the five selected aptamers. To calculate the *K_d_* values, various concentrations of aptamers were incubated with H120 vaccine virus immobilized in the wells of a microtiter plate. The absorbance at 492 nm was determined, and the *K_d_* values were then calculated from the ELAA data using a nonlinear regression equation.

The binding affinity of the selected aptamers to IBV was determined by a direct ELAA, using a coated microtiter plate with vaccine strain H120 virus, followed by incubation of different concentrations of aptamers based on a nonlinear regression equation. The selected aptamers demonstrated affinities in the nanomolar range, where the aptamers Apt_IBV02 and Apt_IBV5 presented the highest affinities (Fig. 1 and Table 1).

### Specificity of aptamers.

**FIG 2:**
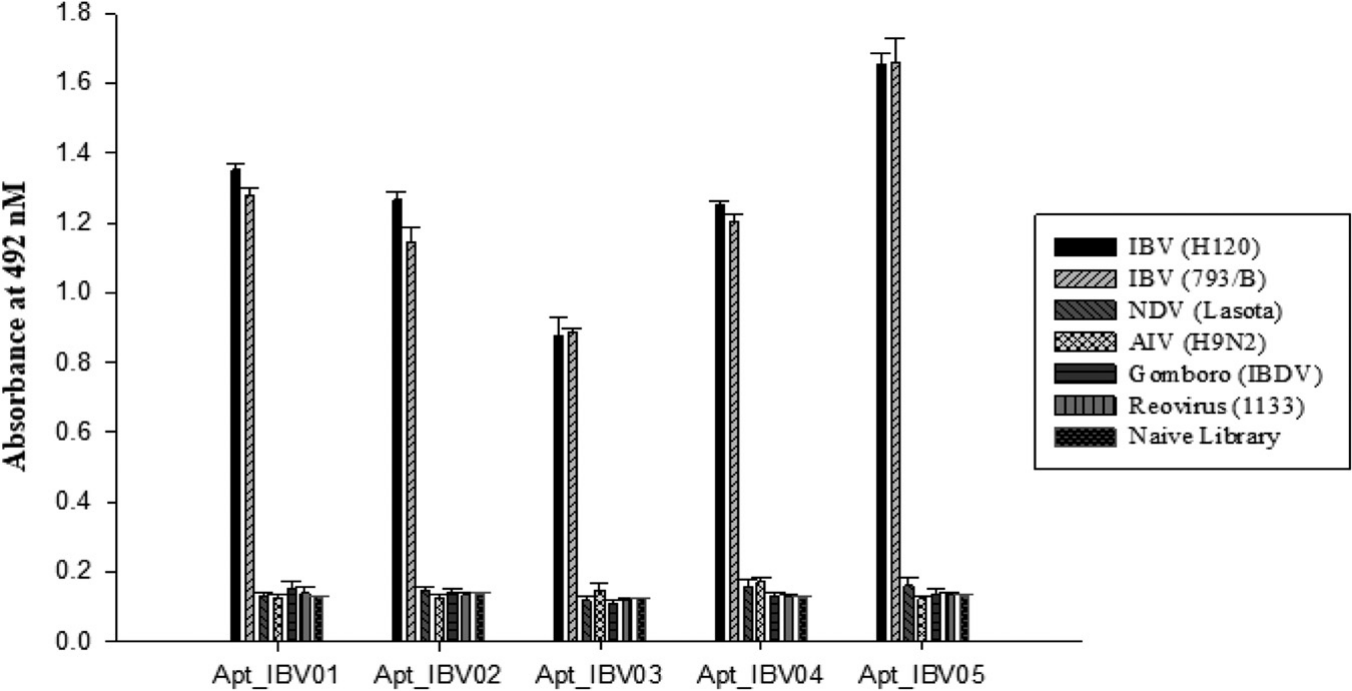
Specificity of the selected aptamers against IBV. The specificity of the five aptamers were tested using ELAA to detect IBV vaccine strains H120-IBV and 793/B, NDV LaSota, IBDV (Gumboro), avian reovirus 1133, or H9N2 avian influenza (AIV). A naive library was used as the negative control. The test was performed in triplicate. Bar graphs show the mean and standard deviation (SD) of the absorbance.

The specificity of the selected aptamers was tested using an ELAA for their ability to bind to two different serotype strains of IBV (H120 and 793/B), different avian viruses (Newcastle disease virus [NDV] LaSota, infectious bursal disease virus [IBDV] [Gumboro], avian reovirus 1133, and H9N2 avian influenza), and a naive library that was used as a negative control (Fig. 2). All five aptamers showed high specificity for the two serotype IBV strains and low background binding levels to the other strains, as well as to the naive library.

### Performance of the aptamers in a sandwich ELAA.

**FIG 3:**
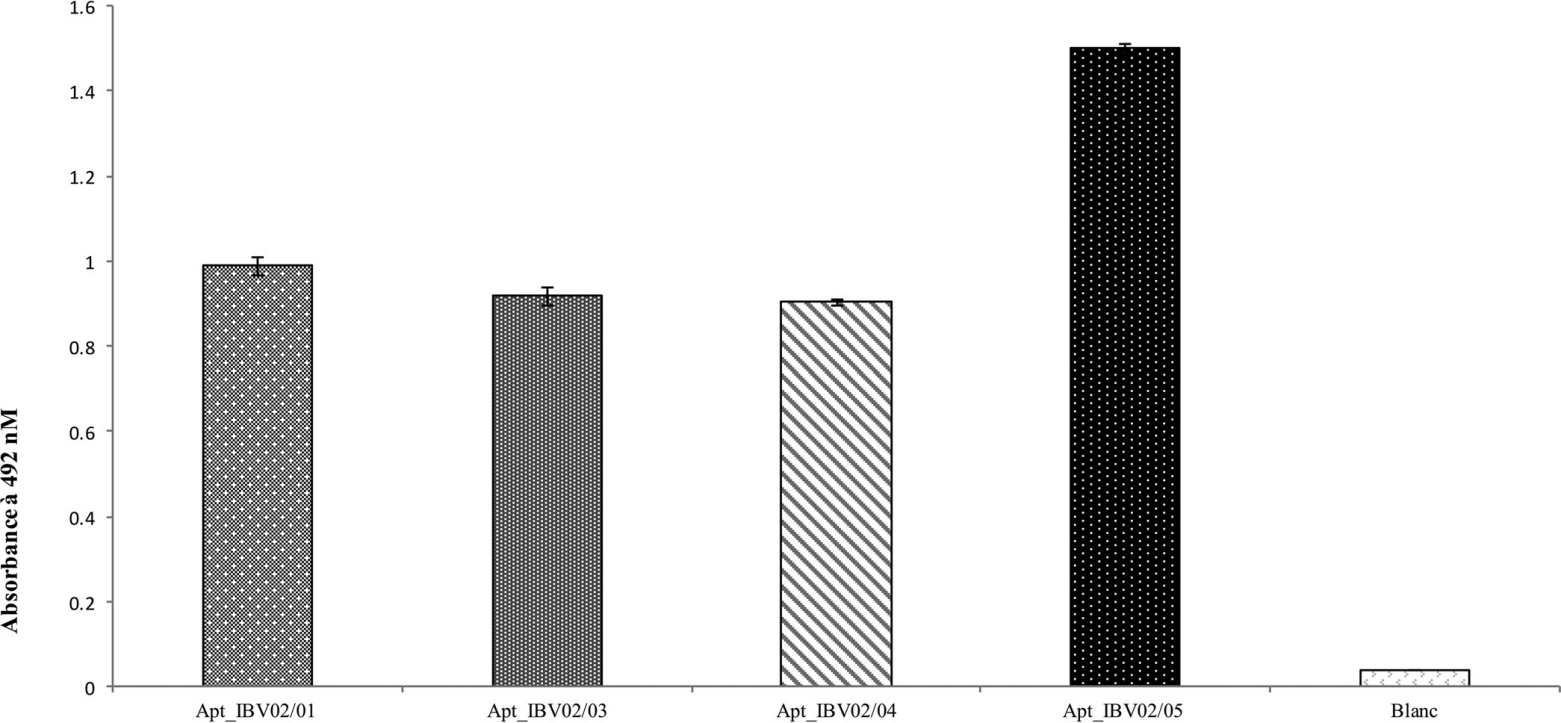
Combined aptamers for a highly efficient sandwich ELAA. Biotinylated Apt_IBV01, Apt_IBV03, Apt_IBV04, and Apt_IBV05 were used as capture binders, while digoxigenin-labeled Apt_IBV02 was used as a reporter in a sandwich ELAA. The reactions were conducted in triplicate, and the absorbance was determined at 492 nm.

To determine the compatibility and best performance for a combination of capture and detector aptamers in a sandwich ELAA, Apt_IBV_02 was coupled to digoxigenin as the detector, while the other aptamers were equipped with a biotin tag to be used for capture. The best performance was observed for the combination of Apt_IBV02 and Apt_IBV_05. Nonetheless, the other combinations resulted in notably higher signals than with the negative control (Fig. 3). To further confirm our results, a competitive test was carried out in which increasing concentrations of Apt_IBV02 resulted in signal saturation, indicating that the two aptamers used for the detection of IBV are not competitive (see Fig. S1 in the supplemental material).

### Secondary structure of the selected aptamers.

The structures of the aptamers Apt_IBV02 and Apt_IBV05 were predicted by means of a free-energy minimization algorithm using the mFold tool, available at http://unafold.rna.albany.edu/?q=mfold (Fig. S2). The results showed that both aptamers have a loop structure in the 5′ end.

### ssDNA aptamer-assisted proximity ligation assay.

The DNA aptamer-assisted PLAs in homogenous and solid-phase formats were designed as described in our previous study, with some modifications (28). The two biotinylated aptamers, Apt_IBV02 and Apt_IBV05, were coupled to two streptavidin-conjugated DNA oligonucleotides to construct PLA probes. The DNA oligonucleotides used in our model were previously empirically evaluated with the PLA technique (25). The simultaneous binding of these two probes with IBV brings the DNA arms into proximity, allowing hybridization to the DNA oligonucleotide connector and subsequent enzymatic ligation. The ligation product will be amplified and quantified by real-time PCR to reflect the number of IBV particles in the sample. In the absence of IBV, no ligation would occur, and hence no detectable signal would be produced.

To determine the performance of the PLAs, we compared the obtained results with those of the sandwich ELAA, as a method for the direct quantification of intact virus, and qRT-PCR, as the gold-standard method, using serial dilutions of the virus and viral RNA, respectively. The results, summarized in Table 2, show that the homogenous PLA and qRT-PCR, with a limit of detection (LOD) of 0.6 EID_50_ (50% egg infective dose)/mL, are 2-fold more sensitive than ELAA. The solid-phase PLA, with an LOD of 0.5 EID_50_/mL, yields the best LOD among the four assays. Furthermore, the solid-phase PLA showed a better dynamic range, upper limit of quantification (ULOQ), lower limit of quantification (LLOQ), and minimal detectable dose (MDD) than the other assays (Table 2). The results show that the PLA, in its two formats, performs well at directly detecting the IBV antigens.

**TABLE 2:** Comparison of various parameters for the assays used in this research for detection of IBV

Parameter	Sandwich ELAA	qRT-PCR	Homogeneous PLA	Solid-phase PLA
LOD (EID_50_/mL)	1.2	0.6	0.6	0.5
LLOQ (EID_50_/mL)	10	0.5	0.3	0.1
ULOD (EID_50_/mL)	7^3^	10^3^	10^4^	10^4^
MDD (EID_50_/mL)	1	0.2	0.2	0.1
Dynamic range	10^3^	10^6^	10^7^	10^8^

### Detection of IBV in field samples.

To evaluate our newly developed sandwich ELAA and PLA techniques, we analyzed 41 field samples taken from poultry suspected of respiratory viral infections. The sandwich ELAA and both the homogeneous and solid-phase PLAs were compared with the qRT-PCR, as the gold-standard assay. The results in Table 3 show that of the 41 suspected samples, only 5 were positive and 36 negative.

**TABLE 3:** Comparison of the sensitivity and specificity of the assays in this work for detection of IBV in 41 field samples^*a*^

Test	No. of samples				Concordance %	Sensitivity		Specificity	
	+/+	+/−	−/+	−/−		TP/(TP+FN)	%	TN/(TN+FP)	%
Sandwich ELAA	5	0	0	36	100	5/5	100	36/36	100
Homogeneous PLA	5	0	0	36	100	5/5	100	36/36	100
Solid-phase PLA	5	0	0	36	100	5/5	100	36/36	100
qRT-PCR	5	0	0	36	100	5/5	100	36/36	100

a TP, true positive (+/+); FP, false positive (−/+); TN, true negative (−/−); FN, false negative (+/−).

**FIG 4:**
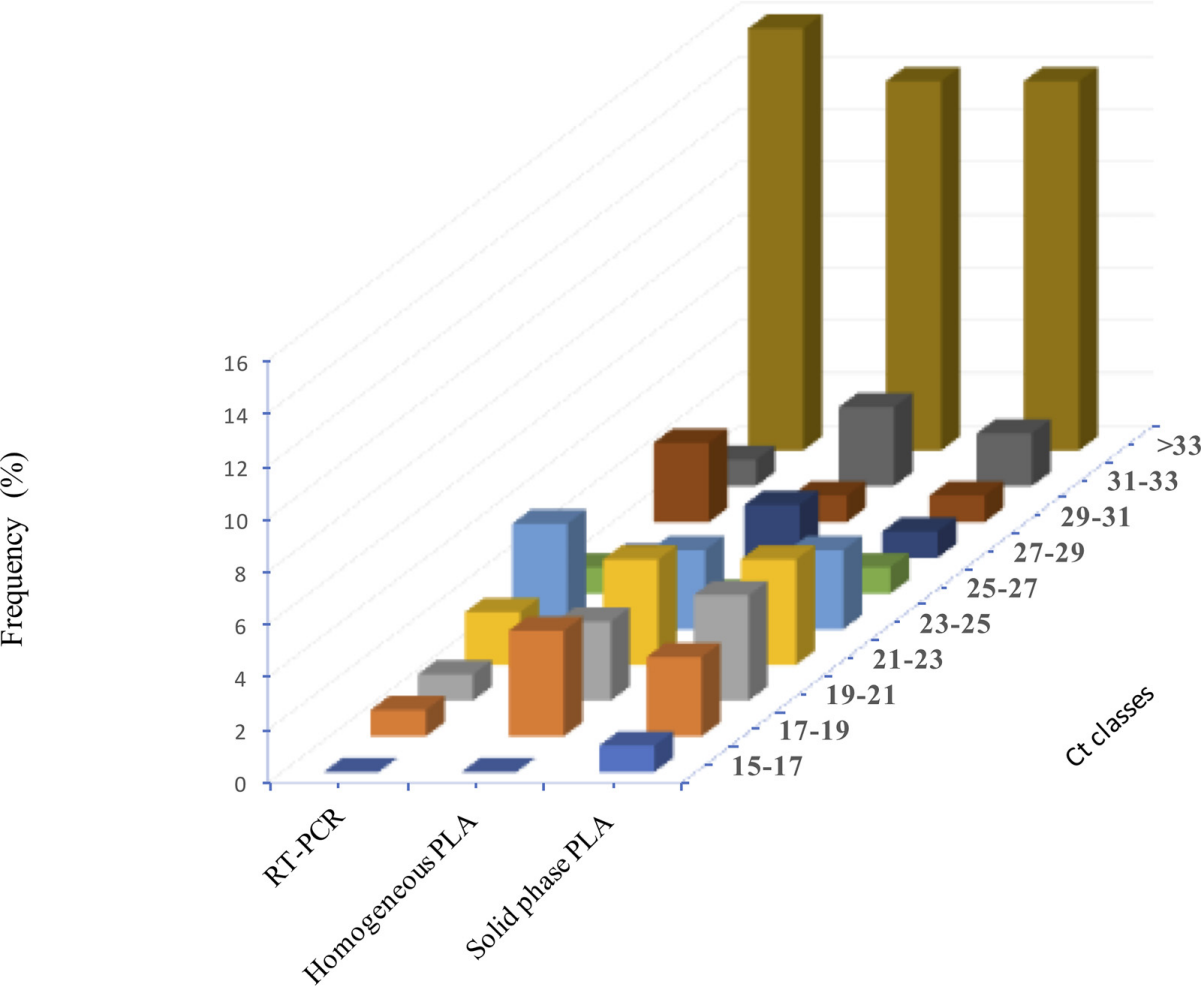
Distribution frequencies of *C_T_* values for a set of 41 field samples analyzed by rRT-PCR and homogenous and solid-phase PLAs for detection of IBV. The sensitivity of the PLA tests in both the homogenous and solid-phase formats was investigated and compared to that of qRT-PCR.

The results obtained using the developed PLA tests are in complete agreement with those determined by qRT-PCR, demonstrating the accuracy of the tests. Detailed statistical analysis relative to the diagnosis of IBV in farm samples is presented in Tables S1 and S2 and was statistically significant (*P* < 0.01). The sensitivity and specificity and comparison of the developed tests with qRT-PCR were also determined and summarized in Table 3. Furthermore, we assessed the distribution frequency of the threshold cycle (*C_T_*) values of qRT-PCR with those of the homogeneous and solid-phase PLAs (Fig. 4). The results showed perfect concordance between all three assays, solidified our conclusions, and confirmed the performance of the developed PLAs.

## DISCUSSION

Infectious bronchitis is a highly contagious and devastating disease, with a very significant economic impact on the poultry industry. To better control and monitor the emergence of this infection, implementation of new, rapid, and accurate diagnostic tests is necessary. Current diagnostic techniques for the detection of avian infectious antigens are more laborious and time-consuming than serological tests and remain limited because of their low specificity and sensitivity (29).

Aptamers have become promising nanomolecules and are of great interest to medical science. Their application has been widely extended from therapeutic molecules to affinity reagents for biosensor development for the detection of a wide range of molecules (30, 31). They are highly specific to their targets, with high affinities in the nano- or subnanomolar range. Furthermore, the production of aptamers is largely economical, reproducible, and relatively easy compared to that of antibodies.

Aptamers are generated using a SELEX process developed by Tuerk, MacDougal, and Gold (32). Over the past 3 decades, multiple variations of SELEX have been reported, including Sweep-CE-SELEX (33), Flu-Mag SELEX (34), CE-SELEX (35), *in silico* SELEX (36) and Capture-SELEX (37). To reduce the processing time and simplify the SELEX procedure, we used the SELEX process based on an NaCl gradient, followed by high-throughput sequencing (26, 27, 38). SELEX by NaCl gradient is a simple and inexpensive approach, allowing the generation of aptamers using very few selection cycles compared to other protocols, which often require up to 10 selection rounds (39). This protocol allows the identification of highly specific aptamers with high affinity through only three rounds of selection. Evaluation of the selected aptamers revealed high specificity and affinity in the nanomolar range, with dissociation constant (*K_d_*) values of 58.2 to 108.61 nm.

The sandwich ELAA was one of the assays used to detect IBV with the developed aptamers, using a combination of Apt_IBV02 and Apt_IBV05, resulting in the detection of the vaccine strain H120 with a LOD of 1.2 EID_50_/mL. Previous works have considered the ELAA as a powerful test for the detection of different viruses (27, 40, 41). However, it does not reach the sensitivity of qRT-PCR as the gold-standard method. Combination of the PLA method with aptamers as affinity binders offers a highly specific and sensitive detection method. PLA has proven to be a robust protein detection method and an exquisitely sensitive technique with a very low background. It was recently used for the detection of NDV and severe acute respiratory syndrome coronavirus 2 (SARS-CoV-2) (28, 42). Our study demonstrates that the homogenous and solid-phase PLAs provide greater sensitivity than the sandwich ELAA and perfect concordance with the qRT-PCR. The sensitivity and LOD of the developed PLA have the same range found in previous work, confirming the robustness of the assay (28). The developed PLA-based tests in this study can be a new tool for the direct detection of pathogens, with high sensitivity. Furthermore, the protocol for the PLA-based tests requires a substantially shorter time compared to the currently used quantitative PCR (qPCR) test, as there is no need for genome extraction, which may take from a few hours to overnight. This approach was validated using field samples, with complete agreement with the currently used qRT-PCR test. The aptamer PLAs established in this study can be extended to the detection of other pathogens with the same sensitivity as qRT-PCR, without any sample preparation steps.

In conclusion, using two complementary aptamers, we have developed rapid and accurate PLA-based diagnostics for the detection of IBV. Both the homogenous and solid-phase PLAs demonstrated higher sensitivity than ELAA, validated using field samples.

## MATERIALS AND METHODS

### Reagents.

Dynabead M-280 streptavidin (10 mg/mL) was purchased from Thermo Fisher Scientific (Artenay, France). All biotinylated aptamers and other DNA oligonucleotides (Table 4) were purchased from RAN BioLinks (Tunis, Tunisia). The washing buffer (PBS-T) was composed of 1× phosphate-buffered saline (PBS; pH 7.2), 0.1% bovine serum albumin (BSA), and 0.05% Tween 20 (Sigma-Aldrich, Taufkirchen, Germany). The PLA buffer was composed of 1× PBS (pH 7.2), 0.1% BSA, 0.05% Tween 20, 100 nM goat IgG, 0.1 μg/μL salmon sperm DNA, and 5 mM EDTA. The probe storage buffer contained 1× PBS (pH 7.2), 0.1% BSA, and 0.05% NaN_3_. The oligonucleotide storage buffer contained 1 mM Tris-buffered saline (Tris-HCl; pH 7.2) and 0.1 mM EDTA. All enzymes and dNTPs (deoxynucleoside triphosphates) were purchased from New England Biolabs (Paris, France).

**TABLE 4:** List of DNA oligonucleotides

Oligonucleotide	Name	Sequence (5′–3′)	Modification	Reference
PCR primers	WP20F1	5′-AGTGCAAGCAGTATTCGGTC-3′	None/5′-biotin	43
	WP20R1	5′-TAAAGCTGATGCGTGATGCC-3′	None	43
Aptamers	Apt_IBV02	5′-CACGTCTCTCTATTCGCTCCCTTCGCTAATTGTTCTCTCC-3′	5′-biotin or 5′-DIG^*a*^	44
	Apt_IBV05	5′-GACTTCCCTCTTTGTGTGTCATTCGGTGTTCTCGCTTTTG-3′	5′-biotin or 5′-DIG	44
Oligonucleotides	SCL1	5′-CGCATCGCCCTTGGACTACGACTGACGAACCGCTTTGCCTGACTGATCGCTAAATCGTG-3′	5′-SAV^*b*^	
	SCL2	5′-TCGTGTCTAAAGTCCGTTACCTTGATTCCCCTAACCCTCTTGAAAAATTCGGCATCGGT-3′	5′-phosphate, SAV-3′	
Ligation template	Connector oligo	5′-TACTTAGACACGACACGATTT-3′	None	
qPCR probe	TaqMan probe	5′-TGACGAACCGCTTTGCTGA-3′	5′-FAM, MGB^*c*^-3′	
qPCR primers	Biofwd (forward primer)	5′-CATCGCCCTTGGACTACGA-3′		
	Biorev (reverse primer)	5′-GGGAATCAAGGTAACGGACTTTAG-3′		
qRT-PCR primers	Forward	5′-GCTTTTGAGCCTAGCGTT-3′	None	45
	Reverse	5′-GCCATGTTGTCACTGTCTATTG-3′	None	45
qRT-PCR probe	TaqMan probe	5′-CACCACCAGAACCTGTCACCTC-3′	5′-Texas red, BHQ-2^*d*^-3′	45

a DIG, digoxigenin.

b SAV, streptavidin.

c MGB, minor groove binder.

d BHQ, black hole quencher.

### Virus strains and clinical samples.

The avian influenza virus isolate A/CK/TUN/145/12 (H9N2) (GenBank accession numbers KP058446 and KP058447 for the HA and NA genes, respectively) was used in the present study. The live vaccine strains H120 and 793/B of avian bronchitis virus and the LaSota vaccine strain of Newcastle disease virus were purchased from a local distributor. Clinical samples from suspected poultry, received for laboratory diagnostics, comprised tracheal (ET) and cloacal (EC) swabs and internal organs (tonsils [TN], livers [L], spleens [S], lungs [LG], and kidneys [K]).

### Selection of aptamers from the ssDNA library.

The protocol for ssDNA aptamer selection was performed as previously described by Hmila et al. and Marnissi et al. (26, 27). A single-stranded aptamer library (WAP40m), consisting of a 40-mer randomized region sequence flanked by constant primer-binding regions, was used (Integrated DNA Technologies, Inc., Coralville, IA). Elution of the binding aptamer was performed using gradient salt (NaCl) elution. The experiment was started by immobilization of the vaccine strain H120 virus on a 96-well ELISA plate. The wells were dry-blotted, 100 μL of single-stranded aptamer library (10 μM) was added, and the plate was incubated for 1 h at room temperature (RT). PBS (100 μL) was then added and incubated for 5 min, and the solution was collected. This was repeated using 0.5, 1.0, 1.2, 1.4, and 1.5 M NaCl, with an incubation period of 5 min each time. The collected 1.5-M NaCl solution was amplified by PCR, using 5 pmol of either WP20F1 or WP20R1 primers (Table 4) and 2× HotStarTaq polymerase (Qiagen, Valencia, CA) with the following steps: 95°C for 15 min, followed by 30 cycles of 95°C for 30 s, 60°C for 30 s, and 72°C for 30 s, and a final elongation of 7 min. Then, asymmetric PCR was performed using 2 μL of symmetric PCR product as the template and biotinylated WP20F1 and WP20R1 reverse primers at a ratio of 25:1. Thermocycling was initiated with a heating step for 5 min at 95°C, followed by 9 preliminary cycles of 95°C for 30 s, 63°C for 15 s, and 72°C for 15 s, 10 subsequent cycles of 95°C for 30 s, 55°C for 15 s, and 72°C for 15 s, and a final step of 72°C for 3 min. The product of the asymmetric PCR was then used to detect the target virus. The vaccine strain H120 virus was immobilized on a nitrocellulose membrane and blocked with 5% skim milk. The product of the asymmetric PCR was added to the nitrocellulose membrane with spotted protein and incubated for 1 h at RT. Thereafter, the membrane was washed with PBS-T, streptavidin-horseradish peroxidase (HRP) conjugate (Sigma-Aldrich) at a 5,000-fold dilution was added, and the membrane was incubated for 1 h at RT. It was then washed three times with PBS-T and developed using West Pico chemiluminescent HRP substrate (Thermo Fisher Scientific). DNA was extracted from the immobilized spots and PCR amplified again by first a symmetric PCR, followed by a second asymmetric PCR, as described above; this screening was repeated three times. The symmetric PCR product from the last screening was then sequenced.

### Identification of aptamers via high-throughput sequencing technology.

Using the primers WP20F1 and WP20R1, the PCR product of the SELEX protocol was purified using a MinElute PCR purification kit (Qiagen). The library was sequenced using the AB library builder system (Thermo Fisher Scientific) and amplified according to the protocol for Ion Xpress Plus and Ion Plus library preparation for the AB library builder system. The library was then purified using the Agencourt AMPure XP reagent (Beckman Coulter). The library size and its concentration were assessed using a Bioanalyzer high-sensitivity chip (Agilent Technologies). The samples were pooled, followed by template preparation on the Ion Chef system, using the Ion PI Hi-Q Chef kit (Thermo Fisher Scientific). The samples were then loaded onto Ion PITM v3 chips and sequenced using the Ion Proton system, with the Ion PITM Hi-Q sequencing 200 kit chemistry (Thermo Fisher Scientific).

### Bioinformatics analysis.

The FASTAptamer toolkit was used, following the steps described by Alam, Chang, and Burke (46). First, the FASTAptamer-Count package was used to rank and sort the sequences by abundance, which was normalized for reads per million (RPM). Second, FASTAptamer-Cluster was used to align and classify the reads by sequence similarity. Only the first five aptamers, from the sorted sequences, were extracted and evaluated for their affinity and specificity.

### Specificity and affinity of the selected aptamers and sandwich ELAA.

The binding affinity of the selected aptamers to IBV and their specificity against different virus strains were tested using a previously established procedure (27).

The sandwich ELAA was performed by immobilizing the different biotinylated aptamers at 10 nM in a streptavidin-coated 96-well plate and incubating the plate for 1 h at RT. The wells were washed three times with 200 μL of washing buffer (PBS-T), 100 μL of vaccine strain H120 in PBS (pH 7.2) was added, and the plate was incubated for another 1 h at RT. The wells were then washed three times with washing buffer, and 1 μM Apt_IBV_02 digoxigenin labeled in 100 μL PBS was used as the reporter. After 1 h of incubation and additional washing, antidigoxigenin antibody (1:2,000) was added to the wells to react for 30 min, followed by three washes. Finally, OPD (o-phenylenediamine dihydrochloride) was added before stopping the reaction by adding H_2_SO_4_ (2N). The absorbance was recorded at 492 nm, and the results of each combination were calculated as the mean ± standard deviation (SD) from three independent experiments.

For the limit of detection (LOD) of the sandwich ELAA, 2-fold serial dilutions of the vaccine strain H120, titrated to 10^5^ EID_50_/mL^−1^, 10 nM biotinylated Apt_IBV05 as the capture aptamer, and 1 μM digoxigenin-Apt_IBV02 as the detection aptamer were used.

### Proximity ligation assays.

**(i) Aptamer-based homogenous PLA.** The PLA probes were constructed by connecting the two biotinylated aptamers (AptIBV05 and AptIBV02) against IBV (Table 1) to the two streptavidin-conjugated DNA oligonucleotides (SCL1 and SCL2) (Table 4) via a biotin-streptavidin interaction. The PLA probe preparation and aptamer immobilization on microplates were carried out as previously described (28). An aliquot of 10^5^ EID_50_/mL^−1^of the vaccine strain H120, diluted in PLA buffer, was used to prepare 2-fold serial dilutions. For each PLA reaction, 45 μL of diluted sample was used. The PLA probe mixtures (aptamer-SLC1 or SLC2 oligonucleotide) were diluted in the PLA buffer to 500 pM. For each homogenous PLA reaction, 2 μL of PLA probe mixture was added to 2 μL of each sample diluted with PLA buffer and incubated for 2 h at RT. For the clinical samples, a positive control comprising 2 μL of diluted H120 vaccine, as well as a negative control containing 2 μL PBS/0.1% BSA, were included. After the incubation, 2 μL of the mixture was added to 25 μL ligation/PCR mix and incubated for 5 min at RT. Then, qPCR was performed using an initial step at 95°C for 2 min, followed by 40 cycles at 95°C for 5 s and 60°C for 30 s. All reactions were carried out in triplicate.

**(ii) Aptamer-based solid-phase PLA.** The biotinylated Apt_IBV02 at 50 nM was used to coat a magnetic bead (100 μL of streptavidin-coated Dynabeads [10 mg/mL] per microplate). The mixture was vortexed to a homogeneous suspension, and 1 μL of the beads was mixed with 45 μL of the diluted samples in PCR strips. The mixture was vortexed and incubated for 1 h at RT with rotation. The magnetic beads were washed three times, the PLA probe mix was added, and the mixture was incubated for 1 h at RT with rotation. The beads were washed twice, 25 μL of ligation/PCR mix was added, and qPCR was performed, as described above. For each run, a positive control containing diluted H120 vaccine and a negative control comprising 0.1% BSA in PBS were included. All washing steps were conducted using the DynaMag-Spin magnet (Thermo Fisher Scientific). To determine the LOD of the PLA, 2-fold serial dilutions of the vaccine strain H120, titrated to 10^5^ EID_50_/mL, were used.

**(iii) qRT-PCR.** One-step rRT-PCR was conducted in a total volume of 15 μL, using the AgPath-ID one-step RT-PCR kit (Applied Biosystems, Artenay, France) by mixing 7.5 μL of 2× RT-PCR buffer, 0.6 μL of 25× RT-PCR enzyme mix, 2 μL of RNA template, 0.3 μM of each forward and reverse primer specific to the polymerase (M) gene, and 0.2 μM TaqMan (Thermo Fisher Scientific) probe and RNase-free water to reach a final volume of 15 μL. The mixture was incubated at 45°C for 10 min, followed by 95°C for 10 min, 45 cycles of 95°C for 15 s, and 60°C for 45 s. The LOD was determined using RNA extracted from 100 μL of the vaccine strain H120, and a half serial dilution was then prepared.

### Statistics.

A one-way analysis of variance (ANOVA) test, using the Simple Interactive Statistical Analysis online tool (http://www.quantitativeskills.com/sisa/index.htm), was used to perform statistical analyses. The results were defined as significantly different at *P* < 0.01. A one-way ANOVA test was also used to calculate the 95% confidence intervals (CIs) of each mean. The mean and the standard deviation (SD), the coefficient of variation percentage (%CV) of the optical density at 492 nm (OD_492_) for the sandwich ELAA, the threshold cycle (*C_T_*) of qRT-PCR, and the PLA data were further analyzed using Excel software. For the qRT-PCR and qPCR readouts, cutoff points were fixed empirically at a *C_T_* value of 40 (47). The relative variability (%CV) between triplicates of each sample was calculated as %CV = (SD of sample triplicates/mean of sample triplicates) × 100 and was defined as significantly different at %CV < 20%. StatPlus Pro v5.9.8 was used to calculate the LOD (46), the LLOQ, the ULOQ, the MDD, and the dynamic range of each method, as described by Marnissi et al. (27).

## References

[1] Collisson EW, Parr RL, Li W, Williams AK. 1992. An overview of the molecular characteristics of avian infectious bronchitis virus. Poult Sci Rev 4:41–55.

[2] Cavanagh D. 2005. Coronaviruses in poultry and other birds. Avian Pathol 34:439–448. doi:10.1080/03079450500367682. 16537157

[3] Wang L, Xu Y, Collisson EW. 1997. Experimental confirmation of recombination upstream of the S1 hypervariable region of infectious bronchitis virus. Virus Res 49:139–145. doi:10.1016/s0168-1702(97)01466-4. 9213388 PMC7126307

[4] Gallagher TM, Buchmeier MJ. 2001. Coronavirus spike proteins in viral entry and pathogenesis. Virology 279:371–374. doi:10.1006/viro.2000.0757. 11162792 PMC7133764

[5] Lewicki DN, Gallagher TM. 2002. Quaternary structure of coronavirus spikes in complex with carcinoembryonic antigen-related cell adhesion molecule cellular receptors. J Biol Chem 277:19727–19734. doi:10.1074/jbc.M201837200. 11912215 PMC8060896

[6] Lai MM, Cavanagh D. 1997. The molecular biology of coronaviruses. Adv Virus Res 48:1–100. doi:10.1016/S0065-3527(08)60286-9. 9233431 PMC7130985

[7] Cavanagh D, Davis PJ, Darbyshire JH, Peters RW. 1986. Coronavirus IBV: virus retaining spike glycopolypeptide S2 but not S1 is unable to induce virus-neutralizing or haemagglutination-inhibiting antibody, or induce chicken tracheal protection. J Gen Virol 67:1435–1442. doi:10.1099/0022-1317-67-7-1435. 3014053

[8] Yamada Y, Liu DX. 2009. Proteolytic activation of the spike protein at a novel RRRR/S motif is implicated in furin-dependent entry, syncytium formation, and infectivity of coronavirus infectious bronchitis virus in cultured cells. J Virol 83:8744–8758. doi:10.1128/JVI.00613-09. 19553314 PMC2738192

[9] Moore KM, Jackwood MW, Hilt DA. 1997. Identification of amino acids involved in a serotype and neutralization specific epitope within the S1 subunit of avian infectious bronchitis virus. Arch Virol 142:2249–2256. doi:10.1007/s007050050239. 9672590 PMC7087143

[10] Johnson MA, Pooley C, Ignjatovic J, Tyack SG. 2003. A recombinant fowl adenovirus expressing the S1 gene of infectious bronchitis virus protects against challenge with infectious bronchitis virus. Vaccine 21:2730–2736. doi:10.1016/s0264-410x(03)00227-5. 12798610

[11] França M, Woolcock PR, Yu M, Jackwood MW, Shivaprasad HL. 2011. Nephritis associated with infectious bronchitis virus Cal99 variant in game chickens. Avian Dis 55:422–428. doi:10.1637/9417-060510-Reg.1. 22017040

[12] Yu L, Liu W, Schnitzlein WM, Tripathy DN, Kwang J. 2001. Study of protection by recombinant fowl poxvirus expressing C-terminal nucleocapsid protein of infectious bronchitis virus against challenge. Avian Dis 45:340–348. doi:10.2307/1592973. 11417813

[13] Terregino C, Toffan A, Beato MS, De Nardi R, Vascellari M, Meini A, Ortali G, Mancin M, Capua I. 2008. Pathogenicity of a QX strain of infectious bronchitis virus in specific pathogen free and commercial broiler chickens, and evaluation of protection induced by a vaccination programme based on the Ma5 and 4/91 serotypes. Avian Pathol 37:487–493. doi:10.1080/03079450802356938. 18798022

[14] Cavanagh D. 2007. Coronavirus avian infectious bronchitis virus. Vet Res 38:281–297. doi:10.1051/vetres:2006055. 17296157

[15] Cavanagh D, Davis PJ, Cook JK. 1992. Infectious bronchitis virus: evidence for recombination within the Massachusetts serotype. Avian Pathol 21:401–408. doi:10.1080/03079459208418858. 18670955

[16] Bourogâa H, Miled K, Gribâa L, El Behi I, Ghram A. 2009. Characterization of new variants of avian infectious bronchitis virus in Tunisia. Avian Dis 53:426–433. doi:10.1637/8666-022609-Reg.1. 19848084

[17] Bourogâa H, Hellal I, Hassen J, Fathallah I, Ghram A. 2012. S1 gene sequence analysis of new variant isolates of avian infectious bronchitis virus in Tunisia. Vet Med (Auckl) 3:41–48. doi:10.2147/VMRR.S32498. 30155432 PMC6065601

[18] Lachheb J, Turki A, Nsiri J, Fathallah I, El Behi I, Larbi I, Ghram A. 2019. Molecular characterization of a unique variant of avian infectious bronchitis virus in Tunisia. Poult Sci 98:4338–4345. doi:10.3382/ps/pez384. 31265109 PMC7107247

[19] Ozalp VC, Eyidogan F, Oktem HA. 2011. Aptamer-gated nanoparticles for smart drug delivery. Pharmaceuticals 4:1137–1157. doi:10.3390/ph4081137.

[20] Kikuchi K, Umehara T, Nishikawa F, Fukuda K, Hasegawa T, Nishikawa S. 2009. Increased inhibitory ability of conjugated RNA aptamers against the HCV IRES. Biochem Biophys Res Commun 386:118–123. doi:10.1016/j.bbrc.2009.05.135. 19501043

[21] Dausse E, Da Rocha Gomes S, Toulmé J-J. 2009. Aptamers: a new class of oligonucleotides in the drug discovery pipeline? Curr Opin Pharmacol 9:602–607. doi:10.1016/j.coph.2009.07.006. 19717337

[22] Fredriksson S, Gullberg M, Jarvius J, Olsson C, Pietras K, Gústafsdóttir SM, Ostman A, Landegren U. 2002. Protein detection using proximity-dependent DNA ligation assays. Nat Biotechnol 20:473–477. doi:10.1038/nbt0502-473. 11981560

[23] Gu GJ, Lund H, Wu D, Blokzijl A, Classon C, von Euler G, Landegren U, Sunnemark D, Kamali-Moghaddam M. 2013. Role of individual MARK isoforms in phosphorylation of tau at Ser^262^ in Alzheimer’s disease. Neuromolecular Med 15:458–469. doi:10.1007/s12017-013-8232-3. 23666762

[24] de Oliveira FMS, Mereiter S, Lönn P, Siart B, Shen Q, Heldin J, Raykova D, Karlsson NG, Polom K, Roviello F, Reis CA, Kamali-Moghaddam M. 2018. Detection of post-translational modifications using solid-phase proximity ligation assay. N Biotechnol 45:51–59. doi:10.1016/j.nbt.2017.10.005. 29101055

[25] Darmanis S, Nong RY, Hammond M, Gu J, Alderborn A, Vänelid J, Siegbahn A, Gustafsdottir S, Ericsson O, Landegren U, Kamali-Moghaddam M. 2010. Sensitive plasma protein analysis by microparticle-based proximity ligation assays. Mol Cell Proteomics 9:327–335. doi:10.1074/mcp.M900248-MCP200. 19955079 PMC2830843

[26] Hmila I, Wongphatcharachai M, Laamiri N, Aouini R, Marnissi B, Arbi M, Sreevatsan S, Ghram A. 2017. A novel method for detection of H9N2 influenza viruses by an aptamer-real time-PCR. J Virol Methods 243:83–91. doi:10.1016/j.jviromet.2017.01.024. 28159667

[27] Marnissi B, Kamali-Moghaddam M, Ghram A, Hmila I. 2020. Generation of ssDNA aptamers as diagnostic tool for Newcastle avian virus. PLoS One 15:e0237253. doi:10.1371/journal.pone.0237253. 32790805 PMC7425888

[28] Marnissi B, Khalfaoui K, Ebai T, Marques Souza de Oliveira F, Ghram A, Kamali-Moghaddam M, Hmila I. 2021. Accurate detection of Newcastle disease virus using proximity-dependent DNA aptamer ligation assays. FEBS Open Bio 11:1122–1131. doi:10.1002/2211-5463.13117. PMC8016122 33595202

[29] Milani G, Fossali EF, Bianchetti MG. 2009. Re: Wang et al.: diagnosis and surgical treatment of nutcracker syndrome: a single-center experience. (Urology 2009;73:871–876). Urology 74:476–477. doi:10.1016/j.urology.2009.04.052. 19646636

[30] Shaban SM, Kim D-H. 2021. Recent advances in aptamer sensors. Sensors 21:979. doi:10.3390/s21030979. 33540523 PMC7867169

[31] Ning Y, Hu J, Lu F. 2020. Aptamers used for biosensors and targeted therapy. Biomed Pharmacother 132:110902. doi:10.1016/j.biopha.2020.110902. 33096353 PMC7574901

[32] Tuerk C, MacDougal S, Gold L. 1992. RNA pseudoknots that inhibit human immunodeficiency virus type 1 reverse transcriptase. Proc Natl Acad Sci USA 89:6988–6992. doi:10.1073/pnas.89.15.6988. 1379730 PMC49630

[33] Mosing RK, Bowser MT. 2009. Isolating aptamers using capillary electrophoresis–SELEX (CE-SELEX), p 33–43. *In* Mayer G (ed), Nucleic acid and peptide aptamers: methods and protocols. Humana Press, Totowa, NJ. 10.1007/978-1-59745-557-2_3 19377982

[34] Stoltenburg R, Reinemann C, Strehlitz B. 2005. FluMag-SELEX as an advantageous method for DNA aptamer selection. Anal Bioanal Chem 383:83–91. doi:10.1007/s00216-005-3388-9. 16052344

[35] Yang M, Peng Z, Ning Y, Chen Y, Zhou Q, Deng L. 2013. Highly specific and cost-efficient detection of Salmonella Paratyphi A combining aptamers with single-walled carbon nanotubes. Sensors (Basel) 13:6865–6881. doi:10.3390/s130506865. 23698275 PMC3690085

[36] Rabal O, Pastor F, Villanueva H, Soldevilla MM, Hervas-Stubbs S, Oyarzabal J. 2016. In silico aptamer docking studies: from a retrospective validation to a prospective case study-TIM3 aptamers binding. Mol Ther Nucleic Acids 5:e376. doi:10.1038/mtna.2016.84. 27754489

[37] Paniel N, Istamboulié G, Triki A, Lozano C, Barthelmebs L, Noguer T. 2017. Selection of DNA aptamers against penicillin G using Capture-SELEX for the development of an impedimetric sensor. Talanta 162:232–240. doi:10.1016/j.talanta.2016.09.058. 27837823

[38] Arnold S, Pampalakis G, Kantiotou K, Silva D, Cortez C, Missailidis S, Sotiropoulou G. 2012. One round of SELEX for the generation of DNA aptamers directed against KLK6. Biol Chem 393:343–353. doi:10.1515/hsz-2011-0253. 22505517

[39] Sola M, Menon AP, Moreno B, Meraviglia-Crivelli D, Soldevilla MM, Cartón-García F, Pastor F. 2020. Aptamers against live targets: is in vivo SELEX finally coming to the edge? Mol Ther Nucleic Acids 21:192–204. doi:10.1016/j.omtn.2020.05.025. 32585627 PMC7321788

[40] Vargas-Montes M, Cardona N, Moncada DM, Molina DA, Zhang Y, Gómez-Marín JE. 2019. Enzyme-linked aptamer assay (ELAA) for detection of toxoplasma ROP18 protein in human serum. Front Cell Infect Microbiol 9:386. doi:10.3389/fcimb.2019.00386. 31799213 PMC6863806

[41] Shin H-S, Gedi V, Kim J-K, Lee D. 2019. Detection of Gram-negative bacterial outer membrane vesicles using DNA aptamers. Sci Rep 9:13167. doi:10.1038/s41598-019-49755-0. 31511614 PMC6739373

[42] Liu R, He L, Hu Y, Luo Z, Zhang J. 2020. A serological aptamer-assisted proximity ligation assay for COVID-19 diagnosis and seeking neutralizing aptamers. Chem Sci 11:12157–12164. doi:10.1039/d0sc03920a. 34123223 PMC8162504

[43] Lamont EA, Wang P, Enomoto S, Borewicz K, Abdallah A, Isaacson RE, Sreevatsan S. 2014. A combined enrichment and aptamer pulldown assay for Francisella tularensis detection in food and environmental matrices. PLoS One 9:e114622. doi:10.1371/journal.pone.0114622. 25536105 PMC4275185

[44] Wise MG, Suarez DL, Seal BS, Pedersen JC, Senne DA, King DJ, Kapczynski DR, Spackman E. 2004. Development of a real-time reverse-transcription PCR for detection of Newcastle disease virus RNA in clinical samples. J Clin Microbiol 42:329–338. doi:10.1128/JCM.42.1.329-338.2004. 14715773 PMC321685

[45] Callison SA, Hilt DA, Boynton TO, Sample BF, Robison R, Swayne DE, Jackwood MW. 2006. Development and evaluation of a real-time Taqman RT-PCR assay for the detection of infectious bronchitis virus from infected chickens. J Virol Methods 138:60–65. doi:10.1016/j.jviromet.2006.07.018. 16934878 PMC7112890

[46] Alam KK, Chang JL, Burke DH. 2015. FASTAptamer: a bioinformatic toolkit for high-throughput sequence analysis of combinatorial selections. Mol Ther Nucleic Acids 4:e230. doi:10.1038/mtna.2015.4. 25734917 PMC4354339

[47] Bustin SA, Benes V, Garson JA, Hellemans J, Huggett J, Kubista M, Mueller R, Nolan T, Pfaffl MW, Shipley GL, Vandesompele J, Wittwer CT. 2009. The MIQE guidelines: minimum information for publication of quantitative real-time PCR experiments. Clin Chem 55:611–622. doi:10.1373/clinchem.2008.112797. 19246619

