# Supplementary material for: Aptamer-Assisted Proximity Ligation Assay for Sensitive Detection of Infectious Bronchitis Coronavirus
Source: Microbiol Spectr. 2023 Jan 18;11(1):e02081-22. doi: 10.1128/spectrum.02081-22 (PMC9927260; doi:10.1128/spectrum.02081-22)
Supplement: Supplemental file 1 — Supplemental material. Download spectrum.02081-22-s0001.pdf, PDF file, 0.2 MB [file spectrum.02081-22-s0001.pdf]

Supplemental Material FOR Publication

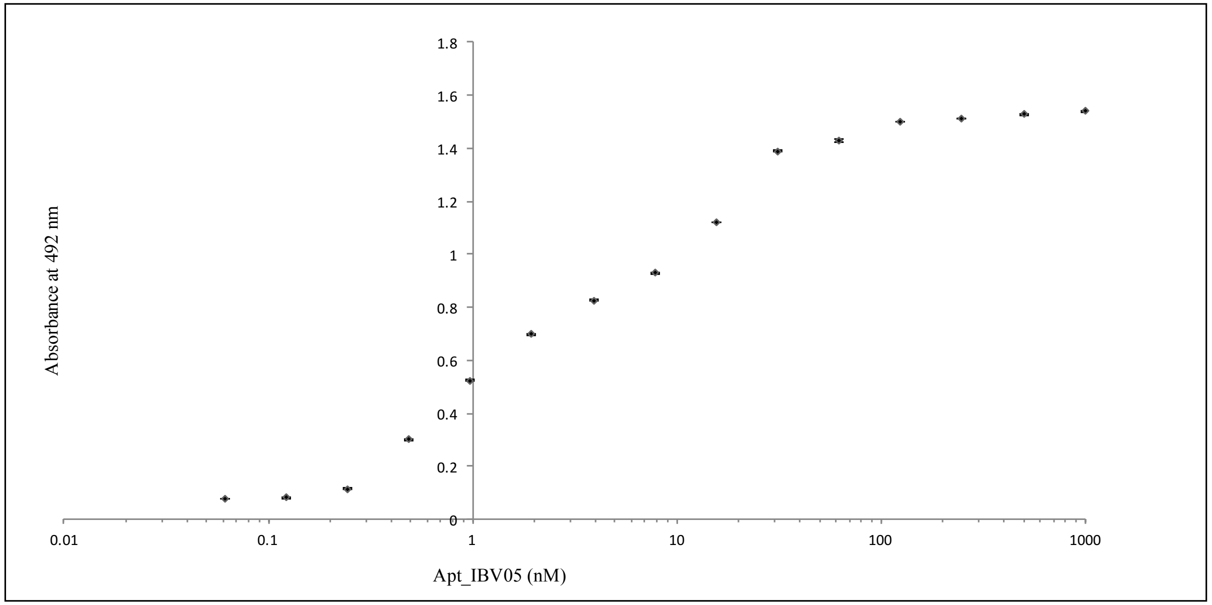

Supplementary Figure 1: Compatibility of the selected aptamers in a sandwich ELAA

A serial dilution of IBV was captured via biotinylated Apt_IBV05 and an increasing amount of digoxygenin Apt_IBV02 was used to detect the virus. The detection curve, with increasing amount of Apt_NDV05, reaches a plateau, indicating that Apt_IBV02 and Apt_IBV05 do not compete.

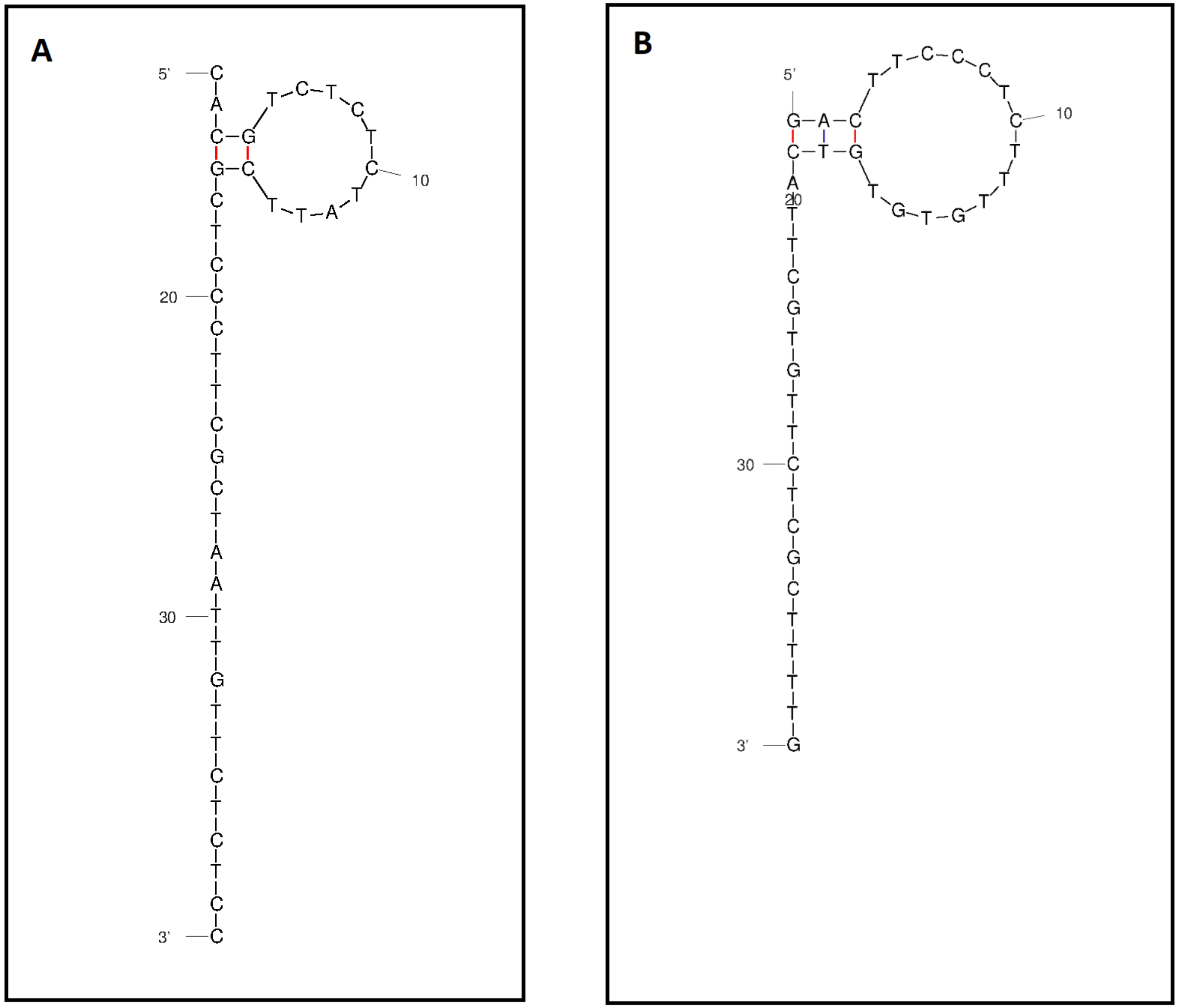

Supplementary Figure 2: Secondary structure of ssDNA aptamers. Secondary structures of Apt_IBV02 (A) and Apt_IBV05 (B), using the mfold software.

The supplemental tables

Supplementary Table 1: Cross-tabulation between Sandwich ELAA and and proximity ligation assay and qRT-PCR
Results of the diagnosis of IBV in tracheal (ET) and cloacal swabs (EC) and internal organs, consisting of allantois (A), kidneys (K), lung (L), liver (Li) and trachea (T) collected from chickens with suspected IBV infection.

	Sandwich ELAA (OD)			qRT-PCR (Ct)			Homogeneous-PLA (Ct)			Solid phase PLA (Ct)		
**Samples**	**Mean OD**	**STDEV**	***p value***	**Mean Ct**	**STDEV**	***p value***	**Mean Ct**	**STDEV**	***p value***	**Mean Ct**	**STDEV**	***p value***
H120 strain	1,761	0,029	0.0001	15,200	0,346	0.0001	15,167	0,289	0	15,133	0,231	0.0001
2321/09	1,527	0,015	0	20,333	0,289	0	20,167	0,289	0.0001	17,967	0,451	0.0001
2322/09	1,602	0,011	0	18,233	0,252	0.0001	18,033	0,058	0	16,933	0,115	0
2325/09	1,388	0,012	0	30,833	0,289	0	30,167	0,289	0.0002	30,033	0,058	0.0001
2330/09	1,258	0,018	0	22,500	0,500	0.0002	21,833	0,289	0.0001	20,833	0,764	0.0003
001/16	0,098	0,010	0.0171	42,000	0,200	0.0456	41,333	0,416	0.026	41,567	0,814	0.1971
003/16	0,088	0,004	0.0143	42,000	0,458	0.0883	41,833	0,289	0.0455	41,667	0,289	0.0506
14/16	0,092	0,005	0.0117	40,500	0,200	0.0066	40,333	0,153	0.0034	40,233	0,252	0.0124
29/16	0,087	0,010	0.037	41,333	0,289	0.0016	41,167	0,289	0.0144	41,033	0,058	0.0117
50/16	0,091	0,003	0.0126	42,167	0,208	0.069	42,100	0,173	0.0121	42,167	0,289	0.5
65/16	0,089	0,002	0.0062	40,967	0,058	0.0044	40,833	0,208	0.0074	40,700	0,265	0.022
95/16	0,101	0,006	0.0436	41,700	0,265	0.0354	41,667	0,208	0.0251	41,533	0,058	0.0349
162/16	0,097	0,002	0.0065	42,233	0,252	0.0962	42,067	0,115	0.0391	42,033	0,058	0.2645
201/16	0,089	0,005	0.0148	42,967	0,252	0.3544	42,900	0,173	0.0506	42,833	0,153	0.0196
003/17	0,100	0,004	0.0148	41,800	0,200	0.0311	41,633	0,153	0.0177	41,533	0,058	0.0349
48/17	0,096	0,003	0.004	41,633	0,153	0.004	41,633	0,153	0.0177	41,633	0,153	0.0336
49/17	0,077	0,004	0.0333	41,833	0,289	0.0066	41,833	0,289	0.0455	41,667	0,289	0.0506
50/17	0,093	0,004	0.0096	41,867	0,153	0.0073	41,833	0,208	0.036	41,700	0,265	0.1403
51/17	0,081	0,006	0.0107	42,933	0,115	0.3374	42,933	0,115	0.0852	42,433	0,404	0.1788
52/17	0,076	0,006	0.0331	42,333	0,306	0.1409	42,333	0,306	0.1452	42,133	0,231	0.4602

54/17	0,094	0,004	0.0086	43,133	0,231	0.208	43,133	0,231	0.0716	43,100	0,173	0.0321
82/17	0,087	0,003	0.0101	41,133	0,321	0.02	41,133	0,321	0.0321	41,133	0,321	0.0492
85/17	0,095	0,004	0.0084	41,667	0,289	0.0039	41,667	0,289	0.1295	41,333	0,289	0.0121
103/17	0,091	0,009	0.01	42,233	0,252	0.0962	42,233	0,252	0.0133	42,233	0,252	0.4248
129/17	0,097	0,009	0.0162	40,633	0,473	0.0166	40,633	0,473	0.0133	40,533	0,451	0.0314
131/17	0,093	0,009	0.0197	41,867	0,231	0.0409	41,867	0,231	0.042	41,700	0,265	0.1485
174/17	0,088	0,007	0.0209	42,167	0,289	0.0237	42,167	0,289	0.1433	42,033	0,058	0.2819
705/17	0,285	0,361	0.2	42,333	0,416	0.1643	42,333	0,416	0.1979	42,233	0,252	0.428
877/17	0,096	0,004	0.0071	40,667	0,493	0.0177	40,667	0,493	0.0145	40,500	0,400	0.0277
29/18	0,092	0,006	0.0024	41,667	0,306	0.0382	41,667	0,306	0.0104	41,633	0,321	0.1208
106/18	0,090	0,009	0.0242	42,333	0,416	0.1643	42,333	0,416	0.2345	42,233	0,252	0.4248
108/18	0,099	0,002	0.3	42,333	0,416	0.1643	42,333	0,416	0.2345	42,233	0,252	0.4248
109/18	0,079	0,003	0.0231	42,933	0,115	0.3374	42,167	0,289	0.1118	42,033	0,058	0.2645
116/18	0,096	0,005	0.0091	42,067	0,115	0.0763	42,067	0,115	0.0391	42,067	0,115	0.3374
134/18	0,093	0,004	0.0093	42,500	0,300	0.2549	42,133	0,115	0.0494	42,100	0,100	0.0207
162/18	0,088	0,008	0.0249	41,433	0,404	0.0341	41,167	0,289	0.0144	41,167	0,289	0.3832
163/18	0,092	0,005	0.0117	42,333	0,153	0.1536	42,067	0,115	0.0391	41,833	0,289	0.1151
215/18	0,104	0,004	0.005	42,233	0,252	0.1468	42,100	0,173	0.0121	42,033	0,058	0.2645
269/18	0,096	0,006	0.002	41,267	0,252	0.0332	41,167	0,306	0.0031	40,867	0,321	0.0329
311/18	0,095	0,006	0.0019	41,867	0,231	0.0716	41,667	0,289	0.0321	41,333	0,289	0.0121
349/18	0,097	0,005	0.0081	42,333	0,289	0.1342	42,133	0,231	0.088	42,067	0,115	0.3466
34/19	1,223	0,027	0.0001	28,333	0,577	0.0007	27,833	0,289	0.0001	26,167	0,289	0
NC	0,064	0,003	-	42,833	0,289	-	42,600	0,173	-	42,167	0,289	-

Supplementary Table 2: Determination of the confidence interval and the coefficient of variability of ELAA sandwich, and PLA and qRT-PCR tests performed to detect IBV in clinical samples

	Sandwich ELAA (OD)			qRT-PCR (Ct)			Homogeneous-PLA (Ct)			Solid phase PLA (Ct)		
Samples	CV%	95% t-C.I		CV%	95% t-C.I		CV%	95% t-C.I		CV%	95% t-C.I	
H120 strain	1,655	1.6886	1.8334	2,279	14.808	15.592	1,903	14.84	15.493	1,526	14.872	15.395
2321/09	0,954	1.4911	1.5635	1,420	20.007	20.66	1,431	19.84	20.493	2,510	17.456	18.477
2322/09	0,687	1.5747	1.6293	1,380	17.949	18.518	0,320	17.968	18.099	0,682	16.803	17.064
2325/09	12.876	1.3584	1.4183	0,936	30.507	31.16	0,957	29.84	30.493	0,192	29.968	30.099
2330/09	1,422	1.2139	1.3028	2,222	21.934	23.066	1,322	21.507	22.16	3,666	19.969	21.698
001/16	9,840	0.074	0.122	0,476	41.774	42.226	1,007	40.862	41.804	1,959	40.645	42.488
003/16	4,749	0.083	0.0924	1,091	41.481	42.519	0,690	41.507	42.16	0,693	41.34	41.993
14/16	5,155	0.0863	0.097	0,494	40.274	40.726	0,379	40.16	40.506	0,626	39.949	40.518
29/16	11,583	0.0759	0.0988	0,698	41.007	41.66	0,701	40.84	41.493	0,141	40.968	41.099
50/16	3,545	0.087	0.0943	0,494	41.931	42.402	0,411	41.904	42.296	0,685	41.84	42.493
65/16	2,247	0.0867	0.0913	0,141	40.901	41.032	0,510	40.598	41.069	0,650	40.401	40.999
95/16	5,782	0.0868	0.1159	0,634	41.401	41.999	0,500	41.431	41.902	0,139	41.468	41.599
162/16	2,139	0.095	0.0997	0,596	41.949	42.51	0,274	41.936	42.197	0,137	41.968	42.099
201/16	5,744	0.0835	0.0951	0,586	42.682	43.251	0,404	42.704	43.096	0,357	42.66	43.006
003/17	3,606	0.0835	0.0951	0,478	41.574	42.026	0,367	41.46	41.806	0,139	41.468	41.599

48/17	3,193	0.0922	0.0991	0,367	41.46	41.806	0,367	41.46	41.806	0,367	41.46	41.806
49/17	5,384	0.067	0.0877	0,690	41.507	42.16	0,690	41.507	42.16	0,693	41.34	41.993
50/17	4,361	0.0826	0.1027	0,365	41.694	42.04	0,498	41.598	42.069	0,634	41.401	41.999
51/17	7,710	0.0655	0.0965	0,269	42.803	43.064	0,269	42.803	43.064	0,952	41.976	42.891
52/17	7,895	0.0611	0.0909	0,722	41.988	42.679	0,722	41.988	42.679	0,548	41.872	42.395
54/17	4,042	0.0843	0.1031	0,535	42.872	43.395	0,535	42.872	43.395	0,402	42.904	43.296
82/17	3,041	0.0804	0.0936	0,781	40.77	41.497	0,781	41.34	41.993	0,781	40.77	41.497
85/17	4,269	0.0846	0.1047	0,693	41.34	41.993	0,693	41.949	42.518	0,698	41.007	41.66
103/17	9,890	0.0686	0.1134	0,596	41.949	42.518	0,596	40.099	41.168	0,596	41.949	42.518
129/17	9,266	0.0749	0.1197	1,163	40.099	41.168	1,163	40.099	41.168	1,112	40.023	41.044
131/17	9,187	0.0718	0.1142	0,552	41.605	42.128	0,552	41.605	42.128	0,634	41.401	41.999
174/17	7,706	0.0714	0.1052	0,685	41.84	42.493	0,685	41.696	42.638	0,137	41.968	42.099
705/17	126,495	0.6113	1.1819	0,983	41.862	42.804	0,983	42.007	42.66	0,596	41.949	42.518
877/17	3,671	0.0869	0.1044	1,213	40.108	41.225	1,213	40.108	41.225	0,988	40.047	40.953
29/18	5,965	0.0787	0.106	0,733	41.321	42.012	0,733	41.321	42.012	0,772	41.349	41.918
106/18	9,493	0.0688	0.1112	0,983	41.862	42.804	0,983	41.862	42.804	0,596	41.949	42.518
108/18	1,538	0.0955	0.1031	0,983	41.862	42.804	0,983	41.862	42.804	0,596	41.949	42.518
109/18	3,349	0.0724	0.0856	0,269	42.803	43.064	0,685	41.84	42.493	0,137	41.968	42.099
116/18	5,225	0.0838	0.1088	0,274	41.727	42.406	0,274	41.936	42.197	0,274	41.936	42.197
134/18	4,301	0.0831	0.1029	0,706	41.942	43.058	0,274	42.003	42.264	0,238	41.987	42.213
162/18	8,637	0.0689	0.1065	0,975	40.976	41.891	0,701	40.84	41.493	0,701	40.84	41.493
163/18	5,451	0.0798	0.1048	0,361	42.16	42.506	0,274	41.936	42.197	0,690	41.507	42.16
215/18	3,990	0.094	0.1147	0,596	41.949	42.518	0,411	41.904	42.296	0,137	41.968	42.099

269/18	6,301	0.0807	0.1106	0,610	40.982	41.551	0,742	40.821	41.512	0,787	40.503	41.23
311/18	5,818	0.081	0.1083	0,552	41.605	42.128	0,693	41.34	41.993	0,698	41.007	41.66
349/18	4,855	0.0856	0.1091	0,682	42.007	42.66	0,548	41.872	42.395	0,274	41.74	42.393
34/19	2,213	1.1554	1.2899	2,038	27.876	28.791	1,037	28.16	27.507	1,103	25.84	26.493
NC	4,749	0.0567	0.0719	0,674	42.507	43.16	0,407	42.796	42.404	0,685	41.84	42.493
